# A local space–time kriging approach applied to a national outpatient malaria data set

**DOI:** 10.1016/j.cageo.2007.05.006

**Published:** 2007-10

**Authors:** P.W. Gething, P.M. Atkinson, A.M. Noor, P.W. Gikandi, S.I. Hay, M.S. Nixon

**Affiliations:** a School of Electronics & Computer Science, University of Southampton, Highfield, Southampton SO17 1BJ, UK; b School of Geography, University of Southampton, Highfield, Southampton SO17 1BJ, UK; c Malaria Public Health & Epidemiology Group, Centre for Geographic Medicine, Kenya Medical Research Institute/Wellcome Trust Collaborative Programme, P.O. Box 43640, 00100 GPO, Nairobi, Kenya; d Department of Zoology, TALA Research Group, Tinbergen Building, University of Oxford, South Parks Road, Oxford OX1 3PS, UK

**Keywords:** Space–time geostatistics, Local kriging, Malaria, Public health, Kenya

## Abstract

Increases in the availability of reliable health data are widely recognised as essential for efforts to strengthen health-care systems in resource-poor settings worldwide. Effective health-system planning requires comprehensive and up-to-date information on a range of health metrics and this requirement is generally addressed by a Health Management Information System (HMIS) that coordinates the routine collection of data at individual health facilities and their compilation into national databases. In many resource-poor settings, these systems are inadequate and national databases often contain only a small proportion of the expected records. In this paper, we take an important health metric in Kenya (the proportion of outpatient treatments for malaria (MP)) from the national HMIS database and predict the values of MP at facilities where monthly records are missing. The available MP data were densely distributed across a spatiotemporal domain and displayed second-order heterogeneity. We used three different kriging methodologies to make cross-validation predictions of MP in order to test the effect on prediction accuracy of (a) the extension of a spatial-only to a space–time prediction approach, and (b) the replacement of a globally stationary with a locally varying random function model. Space–time kriging was found to produce predictions with 98.4% less mean bias and 14.8% smaller mean imprecision than conventional spatial-only kriging. A modification of space–time kriging that allowed space–time variograms to be recalculated for every prediction location within a spatially local neighbourhood resulted in a larger decrease in mean imprecision over ordinary kriging (18.3%) although the mean bias was reduced less (87.5%).

## 1 Introduction

Geostatistical prediction techniques were originally developed for, and remain principally targeted at, spatial-only settings (Chilès and Delfiner, 1999; Goovaerts, 1997; Matheron, 1971). When sampled and unsampled locations are distributed through time as well as space, however, the replacement of spatial-only with space–time geostatistical approaches can offer several benefits including more data to support parameter estimation and prediction and, if present, the exploitation of temporal as well as spatial autocorrelation in observed values. This has led to the development and application of space–time geostatistical models in a range of fields including agricultural (Stein, 1998), atmospheric (De Iaco et al., 2002; Nunes and Soares, 2005) and soil science (Douaik et al., 2005; Snepvangers et al., 2003). Both spatial-only and space–time geostatistical prediction techniques generally rely on the fitting of a random function (RF) model parameterised with a stationary mean and variogram. Where a property of interest displays heterogeneous first- and second-order characteristics, however, alternative non-stationary models may be more appropriate and yield more accurate predictions (Haas, 1995).

In this paper, we take as an example a real-life prediction problem based on a public-health space–time data set from Kenya and develop and implement three different geostatistical prediction methodologies that incorporate a stationary spatial approach, a stationary space–time approach and a locally varying space–time approach in order to compare the accuracy of the resulting predictions.

### 1.1 Case study: the Kenyan health management information system

Increases in the quantity, quality and availability of health data are recognised as fundamental goals in efforts to strengthen health-care systems in resource-poor nations worldwide (AbouZahr and Boerma, 2005; Murray et al., 2004; Stansfield, 2005; WHO/AFRO, 1999). Effective planning and delivery of health system resources requires accurate and timely information on the number of patients visiting health facilities and the types of illness for which they are being diagnosed and treated. Such information requirements are addressed in most countries by some form of National Health Management Information System (HMIS) that coordinates the routine acquisition of treatment records from health facilities and the transfer, compilation and analysis of these data through district, regional and national levels.

Comprehensive HMIS databases rely on prompt monthly reporting from all health facilities. In many resource-poor settings, however, large proportions of health facilities never report or report infrequently, leading to spatially and temporally incomplete national data^1^ (Al Laham et al., 2001; Rudan et al., 2005; WHO/SEARO, 2002). The widespread inadequacy of national HMIS data sets presents a substantial obstacle to evidence-based public health decision making. This problem has led recently to efforts for model health facility utilisation (Gething et al., 2004; Noor et al., 2006) and the development of geostatistical models that aim to predict (i.e. interpolate) the values of missing data within HMIS databases to enable national and sub-national quantification of important public health metrics (Gething et al., 2006).

In this paper, we take the example of the HMIS for Kenya and consider data on malaria proportion (MP), that is, the proportion of the total number of monthly treatment events at each government outpatient facility that result from a diagnosis of malaria. This variable may be of interest for decision makers for priority setting and resource distribution (MoH Kenya, 2001, 2005). Furthermore, predictions of MP may be incorporated into other models to predict the count of malaria cases at facilities (Gething et al., 2006). Sampled and unsampled points in the Kenyan HMIS are distributed at a large number of locations in space (health facilities across the country) and at multiple time periods (months). The spatial structure of MP is determined, in part, by the underlying presence of malaria in the population that is known to exhibit spatial heterogeneity at a range of scales across Kenya (Craig et al., 1999; Omumbo et al., 2005) driven by climatic, topographic and demographic factors.

The objective of this paper is to carry out a series of different geostatistical prediction exercises that predict missing values of MP within the Kenyan HMIS to examine the effect on prediction accuracy of (a) the extension of a spatial-only to a space–time prediction approach, and (b) the replacement of a stationary space–time RF model which requires a single global space–time variogram with a locally varying space–time RF model, which allows the space–time variogram to vary across the study domain.

## 2 Theory

### 2.1 Spatial-only and space–time kriging

Consider a set of spatial data, *z*(**u** *_α_*), of an attribute *z* at *n* locations **u** *_α_*, *α*=1,2,…, *n*, where **u** is a vector of spatial coordinates, {**u**=(*x,y*)}. A standard geostatistical problem is to predict values of *z* at a set of *q* unsampled locations, **u**_0_, z*(**u**_0_), 0=1,2,…, *q*, where the asterisk denotes a prediction. The traditional cornerstone of geostatistics has been the exploitation of spatial correlation between dispersed values *z*(**u** *_α_*) to make these predictions at unobserved points using techniques such as kriging (Matheron, 1971). Along with the data, *z*(**u** *_α_*), kriging predictors require estimates of the covariance between values of *z* separated by different spatial lags, **h**, vectors of distance and direction. These estimates are typically provided by estimating the covariance directly or, more commonly, the semivariance, *γ*, between data pairs at a series of regular lags, taking the average at each lag, and fitting a continuous model to these averages. The variogram model, *γ*(**h**), can then provide semivariance values at any given lag for input into the kriging process.

More recently, this traditional paradigm has been modified to incorporate data distributed through time as well as space (Kyriakidis and Journel, 1999). In this space–time approach, each datum is referenced by its temporal location, *t_α_*, in addition to its spatial location **u** *_α_*, {*z*(**u** *_α_*, *t_α_*); *α*=1,…,*n*}. The space–time variogram is estimated as half the mean squared difference between data separated by a given spatial and temporal lag (**h** *_s_*, *h_t_*):

(1) $${\hat{\gamma }}_{s,t}(h_{s},h_{t})=\frac{1}{2n(h_{s},h_{t})}\overset{n(h_{s},h_{t})}{\underset{\alpha =1}{\sum }}{\left[z(u_{\alpha },t_{\alpha })-z(u_{a}+h_{s},t_{\alpha }+h_{t})\right]}^{2}\text{.}$$

The most commonly used kriging predictor is ordinary kriging (OK). In a space-time framework, this system (space-time ordinary kriging (STOK)) predicts z*(**u**, *t*) as a linear combination of *n*(**u**, *t*) data local in space and time to the prediction location:

(2) $$z_{\mathrm{STOK}}^{*}(u,t)=\overset{n(u,t)}{\underset{\alpha =1}{\sum }}\lambda_{\alpha }(u,t)z(u_{\alpha },t_{\alpha })\mathrm{with}\;\overset{n(u,t)}{\underset{\alpha =1}{\sum }}\lambda_{\alpha }(u,t)=1\text{.}$$

The utility of kriging approaches lies in their ability to determine the weight, *λ_α_*(**u**, *t*), assigned to each neighbouring datum such as to minimise the prediction variance:

(3) $$\sigma_{\mathrm{STOK}}^{2}(u,t)=\mathrm{Var}[z*(u,t)-z(u,t)]$$

while maintaining unbiasedness of the predictor z*(**u**, *t*). In determining the optimum weights, kriging takes into account both the covariances between each datum and the point to be estimated, and the covariances between the data themselves.

### 2.2 Space–time variogram models

A critical stage in the process described above is the choice of model for the variogram or covariance function and the estimation of model parameters. As in the spatial-only case, the principal concerns when modelling space–time autocorrelation structures are to ensure that the model chosen is valid (i.e. that conditionally negative semi-definiteness or positive-definiteness is ensured for variogram or covariance function models, respectively) and that the model is sufficiently flexible to allow fitting to the data though careful estimation of model parameters. While a well-established set of models exists for spatial-only variograms (Deutsch and Journel, 1998), a more diverse range of models have been proposed for the modelling of space-time autocorrelation structures (De Cesare et al., 2001; Kyriakidis and Journel, 1999). These include the product model (Rodriguez-Iturbe and Mejia, 1974), the metric model (Dimitrakopoulos and Luo, 1994), the integrated product model (Cressie and Huang, 1999) and the product–sum model (De Cesare et al., 2001, 2002). This last class of model was adopted for this study because (a) it offers a large class of flexible models that impose less constraints of symmetry between the spatial and temporal correlation components than other classes, (b) it does not require an arbitrary space–time metric to be imposed and (c) the model can be fitted to data using relatively straightforward techniques similar to those established for spatial-only variograms.

The product–sum space–time variogram model, *γ_st_* (**h** *_s_*,*h_t_*), is defined in terms of the separate spatial variogram, *γ_s_*, and temporal variogram, *γ_t_*, as

(4) $$\gamma_{\mathrm{st}}(h_{s},h_{t})=(k_{1}C_{s}(0)+k_{3})\gamma_{t}(h_{t})+(k_{1}C_{t}(0)+k_{2})\gamma_{s}(h_{s})-k_{1}\gamma_{s}(h_{s})\gamma_{t}(h_{t})\text{,}$$

where *C_s_* and *C_t_* are the spatial and temporal covariance, respectively, and *C_s_*(0) and *C_t_*(0) represent the sills (defined as the limit value of each variogram, *γ*(∞)) of the spatial and temporal variograms, respectively. The parameters *k*_1_, *k*_2_ and *k*_3_ are defined as

(5) $$k_{1}=[C_{s}(0)+C_{t}(0)-C_{\mathrm{st}}(0,0)]/C_{s}(0)C_{t}(0)\text{,}$$

(6) $$k_{2}=[C_{\mathrm{st}}(0,0)-C_{t}(0)]/C_{s}(0)\text{,}$$

(7) $$k_{3}=[C_{\mathrm{st}}(0,0)-C_{s}(0)]/C_{t}(0)\text{,}$$

where *C_st_*(0,0) is the ‘sill’ of the space–time variogram *γ_st_*. Several constraints are placed on these parameter values to ensure validity of the space–time variogram (see De Cesare et al., 2001). A key advantage of this model is that *γ_st_* (**h** *_s_*,*h_t_*) is defined entirely in terms of the spatial variogram *γ_s_*(**h** *_s_*), the temporal variogram *γ_t_*(*h_t_*) and the space–time sill *C_st_*(0,0), and that all three can be estimated from the sample space–time variogram surface which is estimated from the data using Eq. (1).

## 3 Data

**Fig. 1:**
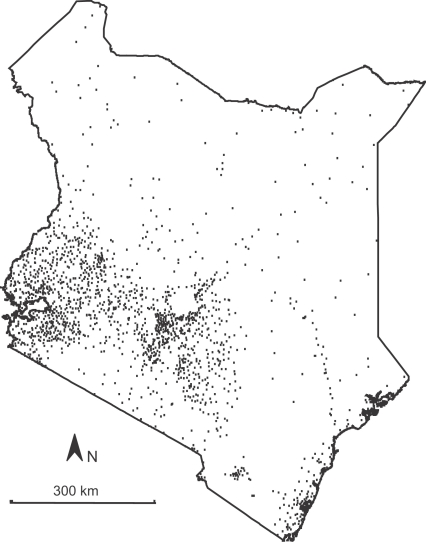
Locations of 1765 outpatient health facilities in Kenya from where malaria proportion data were used in this study. Data represented the monthly proportion of outpatient treatments at each facility for malaria and spanning the period January 1996–December 2002.

Data were obtained from the Department of Health Management Information Systems (HMIS) of the Kenyan Ministry of Health. Data consisted of monthly records from 1765 outpatient departments of government health facilities (Fig. 1 ) over an 84-month period (January 1996–December 2002). Each record included the total number of treatment events made at each facility each month (termed total cases (TC)) and the number of treatment events resulting from a diagnosis of malaria (termed malaria cases (MC)). The variable of interest was the malaria proportion, MP, defined simply as MP=MC/TC. The records were not structured by age, sex or distinguished as initial or follow-up visits, and diagnoses were generally not slide-confirmed. MC therefore represented the count of presumed malaria cases seen as outpatients each month. Data were matched to a georeferenced database, indicating the longitude and latitude of each facility. Details of how this spatial database was constructed are provided elsewhere (Noor et al., 2004) and were updated in 2005 (Noor, 2005). A complete set of 84 monthly records from each of the 1765 facilities would consist of data for 148 260 facility-months. The data set contained data for 63 542 facility-months (43%), meaning 84 718 (57%) were unsampled.

## 4 Methodology

When a disease count is converted to a proportion (e.g. prevalence rate) based on a background denominator value (e.g. the population in a spatial unit), the uncertainty of that proportion can be highly sensitive to the magnitude of the denominator. The effect of TC on MP variance was checked visually (not shown) and found to be minimal, with variance approximately constant for all values of TC. This can be explained by the consistently large TC values (less than 0.2% of TC values were <30 cases) and the fact that malaria is the most common diagnosis, meaning that MC values were generally a substantial proportion of TC. It was decided, therefore, that no aggregation of the monthly MP values was necessary prior to their use in the subsequent prediction exercises.

Three alternative methodologies were used to obtain predictions of MP at individual facility-months in three separate cross-validation procedures. These were OK, STOK and local space-time ordinary kriging (LSTOK). Cross-validation proceeds by the removal of a single datum, *z*(**u** *_α_*,*t_α_*). A kriging prediction, z*(**u** *_α_*,*t_α_*) is then made at this point and the error between datum and prediction is noted. The datum is then replaced, another removed, and the process begins again, eventually repeating for all data to provide a complete set of predicted values for comparison with the data set.

### 4.1 Spatial-only prediction of MP

The full set of *n*=63 542 MP data $\left\{z(u_{\alpha },t_{\alpha });\alpha =1,\ldots ,n\right\}$ was divided by month into {*j*=1,…,*m*} spatial-only sets $\left\{z_{j}(u_{\beta });\beta =1,\ldots ,p(j)\right\}$ where *m*=84 months, and the size of each set, *p*(*j*), varied between months. For each spatial-only set, OK was carried out in the following steps to obtain a set of *p*(*j*) cross-validation predictions $\left\{z_{j}^{*}(u_{\beta });\beta =1,\ldots ,p(j)\right\}$. (1) An omnidirectional sample spatial variogram was estimated from the data using the established method-of-moments approach (Deutsch and Journel, 1998, p. 53). (2) A suitable model was fitted by eye to the omnidirectional variogram from a set of five models, which were the spherical, exponential, Gaussian, power and hole effect models (as defined in Deutsch and Journel, 1998, p. 25). Due to the large number of variograms involved, a parsimonious model structure was adopted for each, consisting of a single-structured model component. The spherical model was selected as offering the best fit to the estimated semivariance values. More importance was attached to ensuring a good fit near the ordinate as values of the variogram at smaller lag separations have more influence in the subsequent kriging. In addition to a spherical component, each model included a nugget component (an intercept on the ordinate of the *y*-axis). The nugget component is used to model the discontinuity caused when semivariance at the very shortest lags does not reach zero. This effect can be caused by various factors, including sampling error and variability in the attribute of interest that is either very short-scale or is not spatially (or, equivalently, temporally) autocorrelated. (3) OK was implemented with the variogram model parameters from (2) to obtain cross-validation predictions $z_{j}^{*}(u_{\beta })$ using the GSLIB *kt3d* routine (Deutsch and Journel, 1998). The search neighbourhood for each prediction consisted of the 50 data closest (using Euclidean distance) to the prediction point.

A single space-time set of *n* cross-validation predictions, $\left\{z_{\mathrm{OK}}^{*}(u_{\alpha },t_{\alpha });\alpha =1,\ldots ,n\right\}$ (subscripted _OK_ to denote prediction using spatial-only OK) was then created by joining each of the *m* spatial-only sets of cross-validation predictions, $z_{\mathrm{OK}}^{*}(u_{\alpha },t_{\alpha })=\cup_{j=1}^{m}z_{j}^{*}(u_{\beta })$.

### 4.2 Space–time prediction of MP

STOK was carried out using the full space-time set of *n*=63 542 MP data $\left\{z(u_{\alpha },t_{\alpha });\alpha =1,\ldots ,n\right\}$ to obtain a set of *n* cross-validation predictions $\left\{z_{\mathrm{STOK}}^{*}(u_{\alpha },t_{\alpha });\alpha =1,\ldots ,n\right\}$ to compare with the *n* data in the following steps. (1) A sample space–time variogram surface ${\hat{\gamma }}_{s,t}(h_{s},h_{t})$ was calculated from the data (Eq. (1)) using a modified space–time GSLIB *gamv* routine (De Cesare et al., 2002). (2) Spatial and temporal variograms were estimated from the space–time variogram surface as ${\hat{\gamma }}_{s,t}(h_{s},0)$ and ${\hat{\gamma }}_{s,t}(0,h_{t})$ by setting *h_t_*=0 and **h** *_s_*=0, respectively (see De Cesare et al., 2001, p. 12). (3) Variogram models were fitted by eye to the separate spatial and temporal sample variograms. As for the spatial-only variograms described above, greater emphasis was placed on ensuring a good fit at smaller lags. Since manual model fitting was required for only one spatial and one temporal variogram, a more complex model structure could be adopted, allowing the use of multiple structured components from the list described above such as to provide a closer fit. The spatial variogram was fitted with a nested model consisting of a nugget, an exponential and a spherical component, and the temporal variogram was fitted with a nested model consisting of a nugget, an exponential and a hole-effect component. (4) The space–time sill, *C_st_*(0,0), was estimated directly from the space–time variogram surface. (5) The space–time sill and parameters from the spatial and temporal variogram models were used to define a product-sum space–time variogram model (Eq. (4)). (6) This variogram model was then used as input in a STOK procedure to obtain cross-validation predictions $z_{\mathrm{STOK}}^{*}(u_{\alpha },t_{\alpha })$ using a modified space–time GSLIB *kt3d* routine (De Cesare et al., 2002). As in the spatial-only case, the search neighbourhood for each prediction consisted of the 50 data closest to the prediction point. The identification of these 50 data required the definition of a space–time distance metric by converting absolute measures of spatial and temporal separation (i.e. kilometres and months, respectively) into relative measures based on their proportion of the maximum spatial and temporal search radii, which were set to 450 km and 84 months, respectively.

### 4.3 Local space–time prediction of MP

The use of STOK, as with OK, implies the adoption of a RF model with stationary mean and variogram. Where first-order heterogeneities exist, the effect on prediction accuracy is often attenuated in practice because each prediction is derived from *n*(**u**, *t*) observations within a limited local space–time neighbourhood *W*(**u**, *t*) centred on the prediction location (**u**, *t*) rather than from all *n* observations throughout the global study domain. As such, the required domain of stationarity for each prediction is reduced to the neighbourhood *W*(**u**, *t*). In the standard form, however, STOK has no such mechanism to attenuate the effects of covariance heterogeneities since it is reliant on the global sample space–time variogram, ${\hat{\gamma }}_{s,t}(h_{s},h_{t})$, which is estimated from all *n* data (Eq. (1)) under the assumption of stationarity. An alternative approach is to adopt a RF model that is *quasi-stationary*, that is, stationarity is considered to exist only within local neighbourhoods (Haas, 1990; Journel and Huijbregts, 1978). This approach was implemented here in a space–time context (denoted as local space-time ordinary kriging (LSTOK)) to obtain a set of *n* local cross-validation predictions $\left\{z_{\mathrm{LSTOK}}^{*}(u_{\alpha },t_{\alpha });\alpha =1,\ldots ,n\right\}$ to compare with the *n* data in the following steps.

(1) The space–time set of *n*=63 542 MP data $\left\{z(u_{\alpha },t_{\alpha });\alpha =1,\ldots ,n\right\}$ were distributed at *l* spatial locations {**u** *_β_*; *β*=1,…,*l*} where *l*=1765, the number of health facilities in the data set. For each of the *l* spatial locations **u** *_β_* where one or more of the *n* cross-validated predictions, z*(**u** *_α_*, *t_α_*), was required, a space–time ‘cylinder’ (Haas, 1995) was defined in which to estimate a spatially local space–time sample variogram, ${\hat{\gamma }}_{s,t}((u_{\beta },u_{\beta }+h_{s}),h_{t})$. Each cylinder consisted of a subset of *n*(**u** *_β_*) data, $\left\{z_{\beta }(u_{c},t_{c});c=1,\ldots ,n(u_{\beta })\right\}$. Each subset was identified as all data located within the nearest *l_c_*=100 locations in space to the prediction location **u** *_β_*, and at any month. The ‘radius’ of each cylinder was therefore equal to the distance from the prediction location **u** *_β_* to its 100th nearest observation in space, and its ‘height’ was *m*=84 months. This approach meant that local neighbourhoods were restricted spatially but not temporally. A balance had to be struck between neighbourhood size (with smaller neighbourhoods considered more appropriate to model as being stationary) and the resulting sample size within each neighbourhood, *n*(**u** *_β_*), with which to estimate each local sample variogram (with smaller subsets resulting in larger uncertainty in the sample variogram). Exploratory analysis of time-series of MP at different spatial locations (not shown) did not suggest the presence of second-order heterogeneity through time. As such, it was decided to include all data through time within each cylinder in order to maximise the sample size *n*(**u** *_β_*) for a given spatially limited neighbourhood.
(2) Spatially local space–time sample variograms were calculated for each spatial location **u** *_β_* using the same procedure as for Section 4.2(1), but applied only to the subset within each spatially local cylinder, $\left\{z_{\beta }(u_{c},t_{c});c=1,\ldots ,n(u_{\beta })\right\}$. After assessing the stability of semivariance estimates at the larger lags, it was decided to model spatial lags up to a maximum of 80% of the diameter of each cylinder and temporal lags up to a maximum of 20 months.
(3) A fitted product–sum space–time variogram model was required for each of the 1765 local variograms. This large number prohibited use of the manual procedure detailed in Section 4.2(3–5) and an automated procedure was developed to replicate these steps. Although estimated and modelled variograms could not be inspected at all 1765 locations, it was necessary to sample the results of the automatic procedure and to make modelling decisions. As such, a set of 50 prediction locations was selected at random and manually checked at each stage. The automatic procedure operated as follows for each local variogram.
  i. Spatial and temporal variograms were estimated from the sample space–time variogram surface as ${\hat{\gamma }}_{s,t}(h_{s},0)$ and ${\hat{\gamma }}_{s,t}(0,h_{t})$ by setting *h_t_*=0 and **h** *_s_*=0, respectively.
  ii. Separate 1-D models were fitted to the spatial and temporal variograms using a weighted-least-squares (WLS) procedure (for brevity, the following description focuses on the spatial variogram, although the equivalent procedure was applied to the temporal variogram). In order to minimise the computational requirements of parameter estimation, and following examination of the 50 monitored local sample variograms, a parsimonious 1-D model consisting of a nugget component and a single spherical component was selected for fitting to all spatial variograms. As such the required parameter set, **θ**, to be estimated for each 1-D model consisted of three parameters (**θ**={*c*_0_,*a*_sph_, *c*_sph_,}), where *a*_0_ is the range parameter of the spherical component and *c*_0_ and *c*_sph_ are the sill parameters of the nugget and spherical components, respectively (Deutsch and Journel, 1998). **θ** was estimated using a nested grid-search algorithm written in ANSI C. The three-parameter 1-D variogram model described above was fitted manually to the spatial variogram estimated from the global space-time sample variogram as described earlier 4.2(1–2) and the resulting parameter set was used as starting values to initialise the algorithm.
    The nested grid-search approach consisted of calculating an objective function, *F*(**θ**), described below, at a set of evenly spaced locations in the 3-D parameter space around the starting values. In the first iteration, *j*=50 values of each parameter were evaluated, meaning objective functions were calculated for *j* ^3^=1.25×10^5^ different parameter sets. The range of parameter values to test in the first iteration was determined heuristically to include a broad swathe of parameter space around the starting values. The range of parameter values was constrained such that impossible values (i.e. *c*_0_<0, *a*_sph_<0, *c*_sph_<0) were not permitted. The parameter set that minimised *F*(**θ**) was identified and became the starting set for the next iteration. Each subsequent iteration evaluated *j* ^3^ evenly spaced parameters over a progressively smaller region of the parameter space, each time identifying the parameter set that minimised *F*(**θ**). The extent to which each iteration converged on progressively smaller regions and the total number of iterations carried out were again determined heuristically, by examining the fit of the resulting models for the 50 monitored variograms.
    The objective function, *F*(**θ**) (Pardo-Iguzquiza, 1999), evaluated for each parameter set was calculated as a weighted sum of squared differences between the spatial variogram, $\hat{\gamma }(i)$, at each of *i*=1,2,…,*n* lags and the value of the variogram model under this parameter set, *γ*(*i*;**θ**):
    (8) $$F(\theta )=\overset{n}{\underset{i=1}{\sum }}w(i)\cdot {\left[\hat{\gamma }(i)-\gamma (i;\theta )\right]}^{2}\text{.}$$
    The weighting scheme used to determine *w*(*i*) was defined as
    (9) $$w(i)=\frac{m(i)}{{\left[\gamma (i;\theta )\right]}^{2}}\text{,}$$
    where *m*(*i*) is the number of data pairs used to estimate $\hat{\gamma }(i)$. In this scheme, each variogram estimate $\hat{\gamma }(i)$ is weighted in approximately inverse proportion to its estimation variance (Cressie, 1985).
  iii. Having estimated the parameter sets for the spatial and temporal variograms, **θ** *_s_* and **θ** *_t_*, the remaining parameter required for the definition of each space–time variogram model was the space–time sill, *C_st_*(0,0). A starting value for *C_st_*(0,0) was estimated from a manual fit of the global space–time variogram, where all the other parameters were provided by **θ** *_s_* and **θ** *_t_* and held constant. The WLS procedure described above was then implemented in the 1-D parameter space to estimate the value of *C_st_*(0,0).
    LSTOK was then implemented to obtain *n* cross-validation predictions $\left\{z_{\mathrm{LSTOK}}^{*}(u_{\alpha },t_{\alpha });\alpha =1,\ldots ,n\right\}$. The kriging algorithm was identical to that used for the global STOK described in Section 4.2 except that, for each prediction, the relevant spatially local space–time variogram model replaced the global model.

### 4.4 Comparison of prediction accuracies

Each of the OK, STOK and LSTOK prediction methodologies described above resulted in a set of *n*=63 542 cross-validation predictions of MP $\left\{z^{*}(u_{\alpha },t_{\alpha });\alpha =1,\ldots ,n\right\}$ to compare with the *n* MP data $\left\{z(u_{\alpha },t_{\alpha });\alpha =1,\ldots ,n\right\}$. In order to compare the performance of the different methodologies, three summary statistics were calculated for each. These were the correlation coefficient between the predicted and actual sets, $\rho [z^{*}(u_{\alpha },t_{\alpha }),z(u_{\alpha },t_{\alpha })]$, the mean prediction error (ME):

(10) $$\mathrm{ME}=\frac{1}{n}\overset{n}{\underset{\alpha =1}{\sum }}z^{*}(u_{\alpha },t_{\alpha })-z(u_{\alpha },t_{\alpha })$$

and the mean absolute prediction error (MAE) (Saito and Goovaerts, 2000):

(11) $$\mathrm{MAE}=\frac{1}{n}\overset{n}{\underset{\alpha =1}{\sum }}\left|z^{*}(u_{\alpha },t_{\alpha })-z(u_{\alpha },t_{\alpha })\right|\text{.}$$

The correlation coefficient provides a straightforward measure of linear association between the data and prediction sets, the ME provides a measure of the bias of the predictor and the MAE provides a measure of the mean accuracy of individual predictions. 2-D histograms were produced to display graphically the bivariate distribution of the data and corresponding predicted values. These plots are more informative than scatter plots when the number of data-prediction pairs is large. Univariate histograms were also produced for each set of prediction errors, $\left\{z^{*}(u_{\alpha },t_{\alpha })-z(u_{\alpha },t_{\alpha });\alpha =1,\ldots ,n\right\}$.

The use of cross-validation as a method of accuracy assessment is limited by a number of factors. Firstly, although each datum is removed temporarily to generate a cross-validation prediction at that point, the variogram is not recalculated with the datum removed and, hence, each cross-validation prediction is not strictly independent of the datum with which it is compared. Where the number of data is large, however, the influence of an individual datum on the sample variogram can be considered negligible. Secondly, the use of simple arithmetic averages to generate estimates of ME and MAE may result in biased estimates when the data are clustered in space and/or time. It is important to distinguish, however, between spatial clustering of the set of facilities and clustering of the data themselves in relation to this background pattern. When an arithmetic average of an attribute at the data locations is used to estimate the mean of that attribute at the unsampled locations, the spatial or spatiotemporal arrangement of the combined set of sampled and unsampled points has no effect on the estimate. Rather, it is the arrangement of the data themselves within this combined set that may introduce bias if they are highly clustered. Although the set of facilities (Fig. 1) are highly spatially clustered, reflecting approximately the spatial distribution of the Kenyan population, the spatiotemporal pattern of data within the set of all points did not display strong clustering either spatially or temporally. The use of cross-validation statistics as comparators of the accuracy of different prediction methodologies further mitigates the effect of the limitations described above, since such effects are consistent between methodologies.

## 5 Results

### 5.1 Variography

**Fig. 2:**
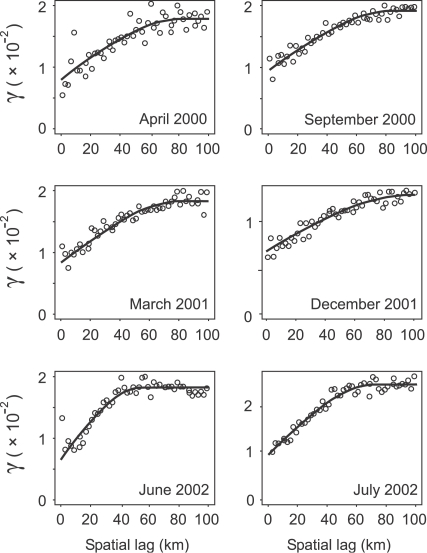
Sample spatial variograms (circles) and fitted variogram models (line) for malaria proportion in six different months during 2000, 2001 and 2002. A total of 84 such variograms were estimated and modelled, one for each month of the study period January 1996–December 2002.

**Fig. 3:**
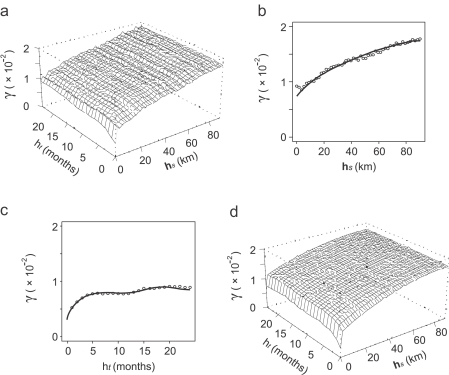
Space–time variography for malaria proportion. Plots shown are (a) sample space–time variogram surface, (b) sample spatial variogram (circles) with fitted 1-D model (line), (c) sample temporal variogram (circles) with fitted 1-D model (line), and (d) 2-D product–sum space–time variogram model. Each vertical axis measures the semivariance, *γ*, and horizontal axes measure either spatial lag (**h***_s_*) or temporal lag (*h_t_*).

**Fig. 4:**
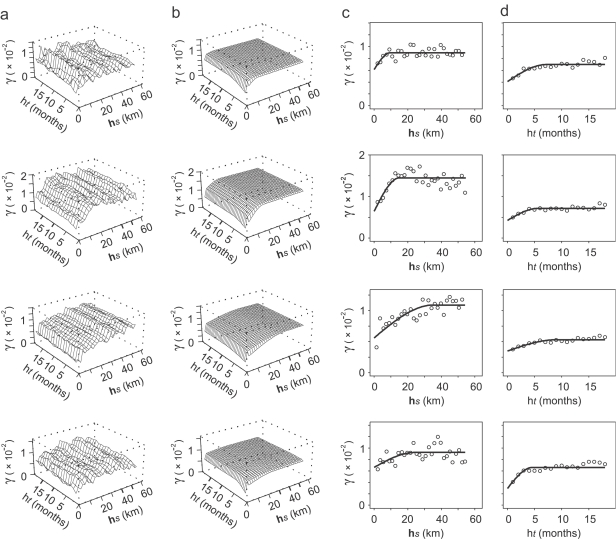
Examples of local space–time variography for four different locations (rows). Variography was carried out automatically in a local neighbourhood around each of 1765 spatial locations where predictions were made. Plots shown for each location are sample space-time variogram surface (column (a)), fitted 2-D product–sum space–time variogram model (column (b)), sample spatial variogram (circles) with fitted 1-D model (line) (column (c)) and sample temporal variogram (circles) with fitted 1-D model (line) (column (d)). Each vertical axis measures semivariance, *γ*, and horizontal axes measure either spatial lag (**h***_s_*) or temporal lag (*h_t_*).

Fig. 2 shows six examples of the spatial variograms that were estimated from spatial-only data for each of the 84 months in the data set, and the corresponding manually fitted variogram models. Sample variogram structure was consistent across the different monthly sample variograms, which supported the use of the same class of variogram model (with a nugget and single spherical component) throughout. The estimated range, sill and nugget parameter values, however, displayed considerable variation between months although no clear patterns could be discerned. Fig. 3 shows the global sample space–time variogram surface and fitted 2-D product–sum model. Also shown are the separate spatial and temporal sample variograms that were estimated using the sample surface, and the corresponding 1-D models. The temporal variogram differed substantially in structure from the spatial variogram, with both a smaller modelled sill value (the sill is the limit value of a transitive variogram, *γ*(∞)) and a smaller nugget-to-sill ratio indicative of greater autocorrelation through time than across space. The spatial variogram shows a small upturn in semivariance for the smallest lags. This effect can be attributed to the nature of facility pairs at these separations. A disproportionate number of these pairs are cross-type: health facilities of the same type are rarely built so close together and it is more commonly the case that large facilities such as hospitals, for example, are surrounded closely by a number of smaller facilities such as health centres or dispensaries. The different facility types are more likely to have different MP values than their spatial separation would otherwise suggest, resulting in a relatively larger semivariance at these short lags. Fig. 4 shows examples for four different locations of the automatic variography procedure implemented to estimate and model local sample space-time variograms for each of the 1765 spatially local neighbourhoods. These four examples illustrate the spatial heterogeneity of the observed space–time autocorrelation structure, with spatial and temporal variogram model parameters varying considerably between the four locations.

### 5.2 Comparison of prediction accuracies

Cross-validation summary statistics for OK, STOK and LSTOK are shown in Table 1 . Both space–time approaches, STOK and LSTOK, resulted in substantially larger values of the correlation coefficient *ρ* than OK (13.1% and 14.8% larger *ρ*, respectively), indicating larger linear correlation between data and prediction sets. ME was small (indicating small overall bias) for all three approaches, although differences between sets were considerable. The value for OK showed the largest bias and those for LSTOK and STOK were substantially smaller (87.5% and 98.4% smaller ME, respectively). The largest MAE was produced by OK predictions, indicating the largest average prediction inaccuracy, with STOK and LSTOK producing more accurate predictions (14.8% and 18.3% smaller MAE, respectively). The overall pattern was that the space–time techniques offered less biased and more precise predictions than OK. Of the two space–time approaches, LSTOK provided more precise predictions than STOK but was slightly more biased overall, although bias was small in both cases.

**Fig. 5:**
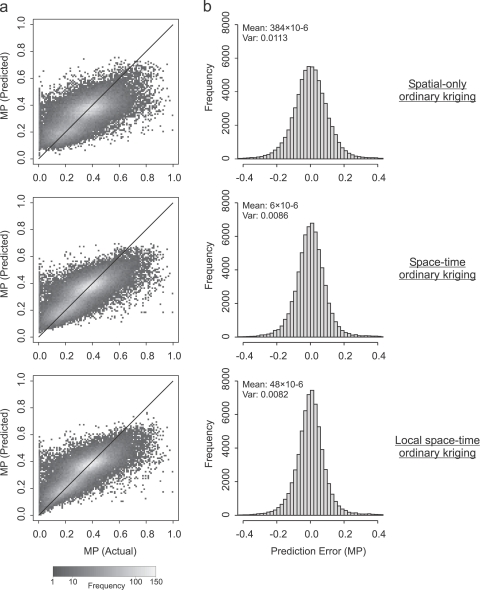
2-D histograms (column (a)) showing bivariate distribution of predicted against actual values for cross-validation predictions of malaria proportion (MP) using three different prediction approaches: spatial-only ordinary kriging, space-time ordinary kriging and local space–time ordinary kriging. Whiter shading represents a higher frequency of values (note non-linear scale). 1:1 line is also provided (diagonal black line) for each plot. Univariate histograms (column (b)) show the distribution of prediction error values for each prediction methodology. Error mean (mean) and variance (Var) are also given.

Fig. 5(a) shows, for each prediction methodology, a 2-D cross-validation histogram illustrating the bivariate distribution of data and prediction sets. The patterns displayed support the summary statistic findings presented in Table 1 and discussed above. A 2-D cross-validation histogram for an accurate prediction exercise would show a high frequency of corresponding data and prediction values along a central region (indicating small imprecision), centred along the 1:1 line (indicating small bias). The 2-D histograms for OK, STOK and LSTOK display progressively tighter central regions, with a greater frequency of values indicated by the whiter shading. Differences in bias are less noticeable, although the progressively smaller bias for OK, STOK and LSTOK for small data values (e.g. <0.1) is clear if the bottom-left corner of each plot is compared. Univariate histograms showing the distribution of error values for each prediction are shown in Fig. 5(b). Errors are approximately Gaussian in each case and the progressively smaller error variances for OK, STOK and LSTOK again correspond to, respectively, more precise predictions.

## 6 Discussion

### 6.1 Comparison of spatial-only and global space–time prediction

When predicting a space–time data set, a potential advantage of the spatial-only approach (e.g. OK) over the global space–time approach (e.g. STOK) is that the spatial variogram is able to vary through time since each month is modelled separately. In contrast, the global space–time variogram averages these individual spatial variograms and month-to-month variability is not represented in the model. This potential advantage of the spatial-only approach is offset by the need to partition the full space–time data set into monthly slices, which may each have insufficient data to obtain a stable estimate of the spatial variogram. A more serious limitation of the spatial-only approach is that any temporal structure present in the data is ignored. The results presented in the previous section showed that the STOK yielded more accurate predictions than OK. The global sample space–time variogram (Fig. 3) displayed substantial temporal autocorrelation and it is intuitive that prediction accuracy should be enhanced by exploiting this temporal structure, allowing predictions to be influenced by observations proximate in time as well as space. A further advantage of STOK over OK in the current context is that the former is significantly less labour-intensive, requiring the estimation and modelling of a single space–time variogram rather than 84 separate spatial variograms. The optimal choice between the two approaches will differ between settings contingent on a range of factors including the space–time distribution of the data and prediction points, and the relative magnitudes of spatial and temporal autocorrelation.

### 6.2 Comparison of global and local space–time prediction

The results described in the previous section showed that more precise predictions were obtained in the space–time prediction exercise when a single global space–time variogram (STOK) was replaced by local space–time variograms that were estimated and modelled for each prediction location using a spatially local subset of data (LSTOK). As with the preceding comparison between OK and STOK, the relative costs and benefits of LSTOK over STOK in the current case may differ in another setting. Where predictions are to be made over a large region displaying second-order heterogeneity, and where data exist at a sufficient density to support stable estimation of variograms within local neighbourhoods, the use of LSTOK offers the potential to provide greater prediction accuracy than STOK, as the current case illustrates. Furthermore, the adoption of an RF model with stationarity of order two or intrinsic stationarity (Journel and Huijbregts, 1978, p. 32) is likely to be more appropriate when these characteristics are considered to exist only within each local neighbourhood rather than throughout the study region.

The principal drawbacks of LSTOK are the difficulties involved in its implementation. Firstly, the calculation of a single sample space–time variogram is computationally expensive (if a spatial variogram is to be estimated at *n*(**h** *_s_*) lags, and a temporal variogram at *n*(*h_t_*) lags, then the equivalent space–time sample variogram requires estimates at *n*(**h** *_s_*)×*n*(*h_t_*) lags). Secondly, where local variograms must be estimated at a large number of locations, automatic variogram model fitting becomes necessary. Although procedures such as WLS allow the implementation of objective criteria for parameterisation, manual fitting is still widely favoured by practitioners of geostatistics as it allows the incorporation of prior knowledge of the phenomena of interest in the variogram model. Algorithms to implement automatic fitting are, again, computationally expensive and can be unreliable, often meaning that variogram models must be parametrically simple, with less structural components than the equivalent manually fitted models. The net effect of using many simple local variogram models compared with a single complex model will clearly depend on several factors including the nature of the global and local spatiotemporal autocorrelation structures being considered and the number of data available with which to estimate local variograms. In the current case, the use of LSTOK over 1765 spatially local neighbourhoods has been shown to offer a modest increase in prediction accuracy over STOK, although at a substantial additional cost in terms of dynamic memory requirements and CPU time.

### 6.3 Predicting malaria proportion

The MP variable, as defined in this study, is dependent on a plethora of (only partly known) epidemiological and facility-specific factors, many of which are likely to vary erratically through space and time. MP is therefore a variable with inherently large uncertainty, and this is reflected in the variograms presented in this study (Fig. 2–4) as large nugget values (the intercept on the ordinate of the variogram model) relative to the structured component (the distance on the ordinate from the intercept to sill). Despite this uncertainty, the presence of spatial and temporal autocorrelation in the MP data justifies the use of geostatistical prediction methods over non-spatial or non-temporal techniques. In a space–time setting such as this, predictions may be required at different levels of spatiotemporal aggregation ranging from, for example, mean national MP over a year down to MP at an individual facility for a specific month. While the space–time approaches presented above provided the least biased predictions, all three methodologies resulted in predictions with a mean bias likely to be negligible for health system planners when, for example, determining the mean malaria proportion for a set of facilities in a given district or province. The importance of the precision of prediction increases as the reporting unit becomes smaller. As such, where predictions are required for individual facility-months, the LSTOK technique would represent the optimal choice, providing predictions at a level of precision likely to facilitate evidence-based decision making.

## 7 Conclusion

The objective of this paper was to implement three different geostatistical approaches that predict missing values of MP within the Kenyan HMIS to examine their relative prediction accuracies. The extension of the established spatial-only approach to a space–time approach yielded substantially more accurate predictions. The further extension of this globally stationary space–time approach to a locally stationary space–time approach whereby space–time variograms were re-estimated for each prediction location within a spatially local neighbourhood yielded a further increase in prediction precision, although was marginally more biased. Space–time approaches implemented here represent a tool that can provide information on an important public-health metric at an accuracy that is likely to be useful to decision makers.

## Figures and Tables

**Table 1:** Comparison of summary statistics for cross-validation predictions of malaria proportion using three different prediction approaches

Modelling approach	*ρ*	ME	MAE
Spatial-only ordinary kriging (OK)	0.6764	0.000384	0.0796
Space–time ordinary kriging (STOK)	0.7651	0.000006	0.0678
Local space–time ordinary kriging (LSTOK)	0.7768	0.000048	0.0650

Statistics shown are correlation coefficient, *ρ*, mean error (ME) and mean absolute error (MAE).

## Footnotes

1 Health Metrics Network, 2005. Issues in Health Information. Available online at http://www.who.int/healthmetrics/library/en/ (accessed 6 May 2006).
